# Promoting Psychosocial Adjustments of Cross-Border Students in Hong Kong: A Resilience and Social Capital Framework

**DOI:** 10.3390/bs14080650

**Published:** 2024-07-27

**Authors:** Qiaobing Wu, Hui Qiu

**Affiliations:** Department of Applied Social Sciences, The Hong Kong Polytechnic University, Hong Kong; qiaobing.wu@polyu.edu.hk

**Keywords:** cross-border children, mental health, life satisfaction, family social capital, community social capital, individual resilience

## Abstract

Nearly 28,000 children, ranging from kindergarten to secondary-school age, commute between mainland China and Hong Kong for education on a daily basis. They are known as cross-border students (CBS)—those who legally hold permanent Hong Kong citizenship and attend schools in Hong Kong, but reside in mainland China, a unique population in the context of cross-border migration. Social media has reported various challenges faced by CBS, but systematic research on this population is limited. This study proposes a resilience and social capital framework to understand the psychosocial adjustments of CBS when faced with different levels of adversities. Using data from a cross-sectional survey of 445 CBS, this study examines how family and community social capital promote the self-esteem, mental well-being, happiness, and life satisfaction of CBS through individual resilience in the face of single and multiple adversities. The results of structural equation modelling suggest that family social capital serves as a significant promotive and protective factor for the self-esteem, mental well-being, happiness, and life satisfaction of CBS in the presence of both single and multiple adversities, while community social capital can promote only mental well-being of CBS in the presence of single or no adversity. Theoretical and practical implications of these findings for researchers, parents, and service professionals are also discussed.

## 1. Introduction

Every weekday morning, nearly 28,000 students leave their homes in mainland China, cross the border for school in Hong Kong, and return home in the afternoon or evening. According to Hong Kong government statistics, during the 2018–2019 school year, there were 27,786 (2031 in kindergarten, 20,188 in primary school, and 5567 in secondary school) of these “cross-border students” [1]. Compared to the surging interest in international and internal migration, not enough attention has been given to this intra-yet-internationally mobile population in the unique geopolitical context of Hong Kong. Earlier studies on cross-border students were based mainly on case studies, observations, and interviews, documenting their social exclusion and other challenges such as the drudgery of commuting daily, language barriers, unfamiliarity with Hong Kong’s educational and political systems, and stigmatization of their legal though second-class citizenship [2,3,4]. Little is known about how cross-border students cope with various difficulties and make positive psychosocial adjustments.

To fill in the gaps, this study proposes an integrated conceptual framework synthesizing social capital theory and the resilience framework to understand the psychosocial adjustments of cross-border students when faced with different levels of adversities. Drawing on a cross-sectional survey of 445 cross-border students in primary and secondary schools in Hong Kong, this study investigates how social capital embedded in family and community contexts shapes the building and functioning of individual resilience, which further enables positive psychosocial adjustments, as measured by four indicators of self-esteem, mental well-being, happiness, and life satisfaction.

The paper starts with an introduction of social capital theory and resilience framework. Political background and previous studies are also mentioned, followed by the proposed conceptual framework and the research hypotheses to be tested. The data, measures, and analysis methods are followed by the results of the descriptive analysis and the structural equation models. The last section presents discussions, conclusions, and implications.

## 2. Literature Review and Conceptual Framework

### 2.1. Individual Resilience, Social Capital, and Psychosocial Adjustments

Since the 1970s, research on resilience has undergone four major waves of evolution and advancement [5], stimulating a growing body of theoretical and empirical work by scholars across multiple disciplines. Despite this explosion of resilience studies, there is still no consensus on a concrete definition of resilience. However, as resilience science and its related fields have gradually matured, two points of agreement, in terms of the conceptualization of resilience, have been achieved. First, resilience is dynamic and multisystemic. Second, resilience has two essential components: adversity and positive adjustments [6,7].

Adversity refers to “disturbances to the function or viability of a system; experiences that threaten adaptation or development” [5] (p. 22). For children and adolescents involved in migration, adversity can be broadly classified into two types: the transition in geographical and sociocultural environment from the sending place to the receiving place, and the transformation and conflicts of personal identity and social roles [8]. Existing studies have well documented the negative and lasting influences of adversity and found that children who were exposed to adversity are at increased risk for a wide set of psychosocial and behavior problems (for example [9,10]). Extending this stream of literature, in the 1970s, scholars began to notice the surprising phenomenon of positive adaptation among some children who functioned and recovered better than others who had been exposed to similar adversity [11,12]. Broadly speaking, resilience refers to the pattern of positive adaptation in the context of past or present adversity. Scholars attempted to identify factors that promoted (in cases of all levels of adversity) or protected (in cases of high levels of adversity) resilience and positive adaptation and reached the consensus that resilient adaptation depends on positive family or community relationships [5,13,14,15,16]. Existing studies provide many criteria for measuring positive adjustments, including but not limited to physical health, psychological well-being, and other dimensions of age-specific competence. Placing children and adolescents in the context of migration, in this study, we follow the findings of Wu and Ou [8] and define resilience as “positive adaptation and development despite the challenging environmental changes and life transitions resulting from migration” (p. 377).

As resilience research evolves, the common understanding of resilience has been extended from a single-system framework to a multi-system framework. Though this study takes the individual as its unit of analysis, we recognize that resilience is subject to the influence of border systems, such as family and community. Treating individuals as active agents who respond and behave within border systems, it is necessary to take into account the attributes inherent in the socioecological systems that are crucial to one’s development. Integrating social capital theory to resilience framework enables examination of how resources in family and community systems shape the building and functioning of individual resilience which further enables individuals’ positive adaptation.

The discussions of social capital stem from Pierre Bourdieu [17] and have been refined and developed by subsequent scholars (for example [18,19,20,21]). Despite scholars’ various interpretations of social capital, one general definition has been achieved: social capital refers to the resources embedded in the social networks accessed and used by actors [22]. As such, social capital has two important features. First, it refers to resources embedded in social relations or social networks rather than possessed solely individuals [22]. Second, it stands for the ability of actors to secure benefits by the access and use of such resources [20].

Scholars have identified two types of social capital—family social capital and community social capital—that are of vital importance to the development of children and adolescents. Family and community social capital stems from the supportive and meaningful interpersonal interactions within families and communities, and both family and community social capital can be classified into two forms: structure and process [23]. For family social capital, structural form refers to the opportunities for interpersonal interactions between parents and children, i.e., family structure; process form emphasizes the quality of parental interactions with their children, i.e., parental support and involvement [23,24]. Similarly, for community social capital, the structural features are attributes that alter the opportunities for interactions between children and adults within the community, such as the physical capacity and residential instability of the community; the process form of community social focuses on the efforts of residents in creating a caring and tightly knit community [23,25]. In this study, we focus on the process forms of family and community social capital. A number of studies suggest that family social capital leads to fewer behavior problems [26], higher educational and occupational aspirations [27,28], better academic achievement [29,30], and more positive psychosocial adjustment [31]. Studies also demonstrated that a higher stock of community social capital, measured by perceived support from neighbors, social cohesion within the neighborhood, and neighbor trusts, is associated with better individual well-being [32], higher levels of health [33,34], increasing academic achievements [23], and lower levels of depression [35].

### 2.2. Political Background and Previous Studies of Cross-Border Students

Hong Kong was a British colony from 1842 to 1997, and China assumed its sovereignty on 1 July 1997. Since then, Hong Kong has been a Special Administrative Region of the People’s Republic of China under the “One Country, Two Systems” principle. Hong Kong is located southeast of mainland China, adjacent to Guangdong Province. Although Hong Kong is part of China, a physical border exists between mainland China and Hong Kong, and travelers must pass through both outbound and inbound immigration checks. This constitutes a clear boundary between two distinct but closely connected economic, social, and political systems.

This peculiar geopolitical context gives rise to a unique mobile population: cross-border students [36]. As of the 2018–2019 school year, a total of 27,786 “cross-border students”, ranging from kindergarten to secondary school, resided in mainland China but attended school in Hong Kong [1]. Cross-border students can be roughly categorized into two types. The majority were born of marriages between mainland Chinese females and Hong Kong males, which is the predominant combination of cross-boundary marriages. This type is called “single-not” (*danfei*) children, as one of their parents is not a Hong Kong resident. To be reunited with their husbands in Hong Kong, mainland wives have to apply and wait for approximately four years before receiving a one-way permit issued by the relevant authorities of the Public Security Bureau in mainland China. While some cross-border families then move to Hong Kong (after the mother has obtained Hong Kong residency), others remain in mainland China for reasons such as social networks, lower costs of living, and better housing environments or work opportunities.

A small number of cross-border students have two mainland-born parents. This second type is called “double-not” (*shuangfei*) children, as neither of their parents is a Hong Kong resident. In July 2001, the Court of Final Appeal ruled that babies born in Hong Kong to Chinese nationals have the right of abode in Hong Kong. Increasing numbers of mainland families then began giving birth to children in Hong Kong as a strategy to avoid the PRC’s one-child policy and/or to secure Hong Kong citizenship, seen as a path to a better future. This burgeoning phenomenon of birth tourism quickly led to protests by locals over the resulting shortage of obstetric services and school placements. On 1 January 2013, the Hong Kong government banned mainland birth tourism, and implemented a zero-quota policy on obstetric services for mainland women whose spouses are not Hong Kong residents. The number of children born in Hong Kong to mainland parents, which had surged significantly from 620 in 2001 to 35,736 in 2011, fell to just 393 in 2019 [37].

As mentioned earlier, almost all cross-border students have been born in Hong Kong and legally hold Hong Kong citizenship. Though cross-border families choose to reside in mainland China for various reasons, these cross-border students are not legally household members (under the *hukou* household registration system) and are therefore ineligible to attend public school. Thus, whether these cross-border families like it or not, cross-border schooling seems to be their only choice. Studies have attempted to understand the challenges faced by cross-border students. The biggest challenge is the daily transportation time [3]; some cross-border students spend four hours a day commuting [38]. A related consequence is limited opportunities to participate in extracurricular activities [39]. Differences in language and in the educational and political systems also lead to communication difficulties and identity confusion [3,4]. Even though cross-border students hold legal citizenship in Hong Kong, local media and people still tend to stigmatize them as socially illegitimate [40,41]. Most studies on cross-border students have drawn on qualitative analysis and are typically small-scale. Also, because these existing studies mainly focus on these students’ perceived roles in Hong Kong, their mental health and attitudes towards life are typically not considered, especially in terms of subject measurements of mental health outcomes in larger samples.

### 2.3. Conceptual Framework and Research Hypotheses

**Figure 1:**
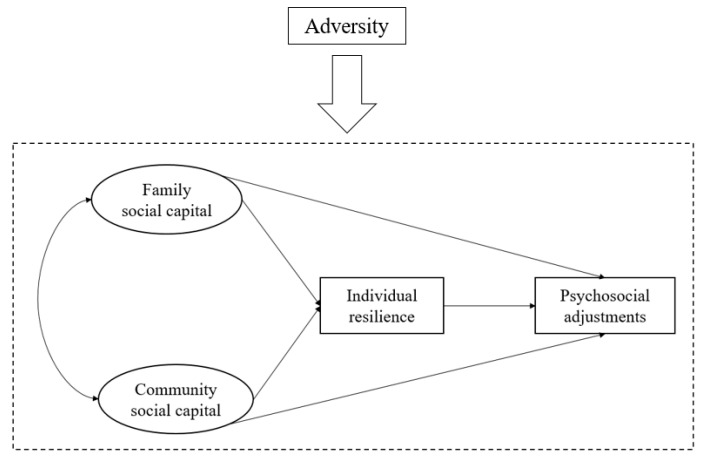
Conceptual framework.

In light of these existing studies and their knowledge gaps, this study synthesizes resilience and social capital theories, and proposes a new conceptual framework to understand the psychosocial adjustments of cross-border students. As shown in Figure 1, in the presence of adversity which varies in extent, family and community social capital act as two key interrelated contexts for building and enhancing individual resilience, which further enables cross-border students’ positive adaptation (self-esteem, mental well-being, happiness, and life satisfaction). Family and community social capital also play a significant role, directly influencing the psychosocial adjustments of cross-border students.

From the rationales mentioned above, the present study derives three major hypotheses to be tested. First, this study expects that social capital embedded in family and community context has direct and positive effects on cross-border students’ psychosocial adjustments (*Hypotheses 1a & 1b*). Second, this study anticipates that family and community social capital exert positive influence on individual resilience, which further promotes psychosocial adjustments (*Hypotheses 2a & 2b*). Third, adversities are unlikely to impact the functioning and building of individual resilience equally [42], and it is anticipated that the proposed framework works differently when levels of adversity are different. It is expected that the total effects of family and community social capital on cross-border students’ psychosocial adjustments are stronger when they are facing multiple adversities (*Hypotheses 3a & 3b*).

## 3. Data and Methods

### 3.1. Data and Sample

The data used in this study were obtained in a large-scale cross-sectional survey in Hong Kong following a school-based multi-stage cluster sampling procedure. As the survey focused on the education and mental health of Hong Kong students from different immigration backgrounds, three districts from the New Territories and Kowloon areas which had relatively higher percentages of immigrant students were selected. According to information provided by the Education Bureau of the Hong Kong Government, two primary schools and two secondary schools were randomly selected within each district. For each selected primary school, two classes from the 4th to 6th grades were randomly selected. For each selected secondary school, two classes from the 7th to 9th grades were randomly selected. All students in the selected classes were invited to complete the survey. In total, 2180 students were recruited in the survey, including 445 cross-border students, 348 new-immigrant students (students who were born in mainland China, of Chinese nationality, and have stayed in Hong Kong for less than seven years), and 1387 local students. The survey was conducted in 2016–2017 and collected information such as personal and family demographics, family and community relationships, and multiple education and mental health indicators. Cross-border students were further invited to complete a short questionnaire on their daily border-crossing experiences. In this study, the sample is restricted to 445 cross-border students. 

The survey was conducted in classrooms, and research-team members were present to address questions or concerns students might have about the survey. Both students and parents signed consent forms before the survey. This study was approved by the Ethics Review Committee of the first author’s institution.

### 3.2. Measures

#### 3.2.1. Psychosocial Adjustments 

Four indicators of cross-border students’ psychosocial adjustments were measured as dependent variables as follows: self-esteem, mental well-being, happiness, and life satisfaction. Self-esteem was measured by the Chinese version of the classic 10-item Rosenberg Self-Esteem Inventory (RSEI) [43]. Students rated themselves on statements like “I feel that I am valuable”, with a scale ranging from 1 (strongly disagree) to 4 (strongly agree). These 10 items were summed up in analysis, and higher scores represented higher levels of self-esteem. Mental well-being was measured by the 14-item Warwick-Edinburgh Mental Well-being Scale (WEMWBS) [44]. Responses to each item varied from 1 (never) to 5 (always). These 14 items were summed up in analysis, and higher scores indicated higher degrees of mental well-being. Happiness was measured by one single-item question, namely “Overall, do you feel happy?”. Answers to this question ranged from 1 (not happy at all) to 5 (extremely happy). Life satisfaction was measured by the 5-item Satisfaction with Life Scale (SWLS, [45]). Students rated themselves on statements like “I am satisfied with my life”. Responses varied from 1 (strongly disagree) to 7 (strongly agree). The sum of these 5 items was used in analysis, and higher values indicated higher levels of life satisfaction. Cronbach’s alpha for self-esteem, mental well-being, and life satisfaction in this study was 0.803, 0.961, and 0.915, respectively. 

#### 3.2.2. Individual Resilience, Family and Community Social Capital

Individual resilience was measured on a 15-item scale. Students rated themselves on statements like “I have confidence in myself that helps me to overcome adversities” and “I can always handle things well, no matter how”, with responses ranging from 1 (strongly disagree) to 7 (strongly agree). The sum of these 15 items was used in analysis, and higher scores indicated higher levels of individual resilience. Cronbach’s alpha for individual resilience in this study was 0.969.

Family social capital consisted of five aspects: parent–child interaction, parental monitoring, parents’ knowledge of children’s after-school whereabouts, relationship with mother, and relationship with father. Cronbach’s alpha for parent–child interaction, parental monitoring, parents’ knowledge of children’s after-school whereabouts, relationship with mother, and relationship with father was 0.749, 0.727, 0.865, 0.766, and 0.825, respectively.

To reflect parent–child interaction, eight questions were used, asking whether the student and parents had undertaken certain activities together in the past month, namely going shopping, exercising, watching TV, playing together, discussing things happened in school, discussing things that made the student sad, discussing people that the student and parents both knew, and visiting relatives/friends. Answers were recorded as 1 if the student reported having carried out the mentioned activity with his/her parents in the past month, and were recorded as 0 if otherwise. The sum of these eight items was used in the analysis, and higher scores demonstrated higher levels of parent–child interaction. 

Parental monitoring was measured by eight questions on parental attendance at school and parental supervision at home, such as “Do your parents often attend the parent-school meeting?” and “Do your parents often check if you have finished your homework?” with answers varying from 1 (never) to 4 (often). Answers to these eight items were summed up in the analysis, and higher values implied higher levels of parental monitoring.

Four questions were asked to measure parents’ knowledge of students’ after-school whereabouts—“Do your parents know where you are/who you are staying with/what you are doing/when you will come home after school?”—with responses varying from 1 (never) to 5 (always). The sum of these 4 items was used in analysis, and higher scores indicated higher levels of parents’ knowledge of students’ after-school whereabouts. 

Students’ relationship with mother/father was measured using a 12-item scale, a shortened version of the Inventory of Parent and Peer Attachment Scale (IPPAS, parent subscale) [46]. Students were asked to evaluate their relationships with their parents on a five-point scale varying from 1 (never) to 5 (always), with items such as, “I tell my mother/father about my problems and troubles” and “My mother/father respects my feelings”, etc. The sum of the 12 items were included in the analysis, and higher scores denoted a better relationship with their mother/father.

Community social capital consisted of four aspects: neighborhood caring, social cohesion and trust among adults, social cohesion and trust among children, and sense of belonging to the community. Neighborhood caring was measured by one single-item question, namely “Do neighbors care about you?” Answers ranged from 1 (not at all) to 5 (very much). The latter three aspects of community social capital were assessed by an 18-item scale, with items such as, “Neighbors in the community are always helpful” and “I feel that I am a member of this community”. Students rated themselves on these statements, with the scale ranging from 1 (strongly disagree) to 5 (strongly agree). Responses were summed up for each aspect, and higher values meant higher levels of community social capital. Cronbach’s alpha for social cohesion and trust among adults, social cohesion and trust among children, and sense of belonging to the community was 0.743, 0.534, and 0.920, respectively.

#### 3.2.3. Adversity

Cross-border students were invited to answer some supplementary questions on their daily border-crossing experiences. Fourteen questions were used to represent the adversity experienced by cross-border students. Students rated themselves on the difficulties they encountered, such as “Physically tired and lack of sleep”, “Difficult to adapt to the course”, and “Unable to attend extra-curriculum activities in school”, with the scale ranging from 1 (not difficult at all) to 5 (extremely difficult). Responses to each item were then recorded as 0 if the answers were “1—Not difficult at all”, “2—A little difficult”, or “3—Not sure”, and recorded as 1 if the answers were “4—Very difficult” or “5—Extremely difficult”. The sum of these 14 recorded items was used in analysis, and higher values indicated higher levels of adversity. Cronbach’s alpha for adversity in this study was 0.884. In other words, adversity measures the total number of items that cross-border students experienced and found to be very or extremely difficult for them. In later analysis, we allocated cross-border students into two groups based on the value of adversity. Cross-border students who scored 0 or 1 were classified into the single or no adversity group (SA), and those who scored 2 or above were classified into the multiple adversities group (MA).

#### 3.2.4. Control Variables

The control variables were students’ gender (recorded as 1 for male and 0 for female), student’s age (in years), duration of being a cross-border student (in years), two dummies indicating whether the father and mother had obtained associate degree or above, and two more dummies denoting whether the student lived with their father or mother.

### 3.3. Data Analysis

Structural equation modeling (SEM) was employed for analysis, for two major reasons. First, SEM enables the construction of latent variables and permits linkages between latent and observed variables. Second, SEM overcomes the limitation of conventional regression methods, allows for correlation between family and community social capital, and can explore how family and community social capital simultaneously contribute to cross-border children’s individual resilience, which further affects their psychosocial adjustments.

There were three steps undertaken in the analysis. First, a measurement model was applied to construct the two latent variables, namely family and community social capital. Second, SEM was estimated on four different outcome variables (specifications shown in the conceptual framework) to test hypotheses 1 and 2. Third, the sample was split into two groups according to the total number of adversities experienced by the students. A multiple-group analysis was further performed to test hypothesis 3, which estimated models for two subgroups: the single or no adversity group (SA), and the multiple adversities group (MA). Adversities and control variables were included as covariates in SEM for both full and sub-group samples. Full information maximum likelihood (FIML) was employed to handle missing data. All analysis was performed using STATA16.

## 4. Results

### 4.1. Descriptive Statistics

Table 1 provides the percentages and means of variables included in the analysis. On average, cross-border students scored 29.295, 51.440, 3.831, and 25.100 for self-esteem, mental well-being, happiness, and life satisfaction, respectively. As for the daily commuting experience between mainland China and Hong Kong, on average, cross-border students suffered from nearly two types of difficulties. Overall, 52.871% of the cross-border students were male, and the average age was 11.108 years old. Only 12.557% and 8.716% of their fathers and mothers had finished associate degree or above, respectively. In total, 82.883% of cross-border students lived with their fathers at home, and 95.045% of them lived with their mothers at home. The average duration of being a cross-border student was 5.235 years, with a standard deviation of 2.483.

### 4.2. Results of Measurement Model

Appendix A presents the standardized and unstandardized factor loadings of all indicators. All observed variables were significantly loaded to the latent constructs, and all standardized factor loadings were above 0.5. The measurement model showed good model fit (CFI  =  0.972, TLI  =  0.960, RMSEA  =  0.056).

### 4.3. Results of SEM Analysis

**Figure 2:**
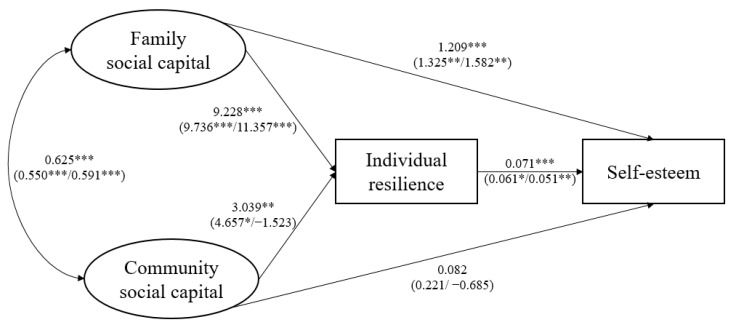
SEM results for self-esteem. * *p* < 0.1, ** *p* < 0.05, *** *p* < 0.01.

**Figure 3:**
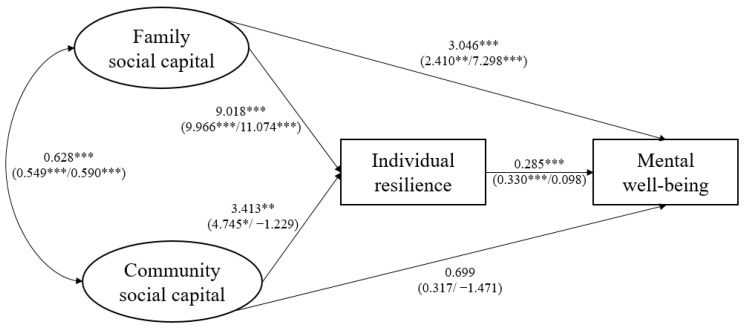
SEM results for mental well-being. * *p* < 0.1, ** *p* < 0.05, *** *p* < 0.01.

**Figure 4:**
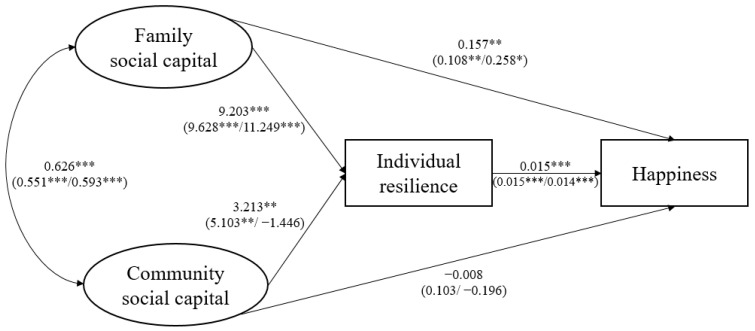
SEM results for happiness. * *p* < 0.1, ** *p* < 0.05, *** *p* < 0.01.

**Figure 5:**
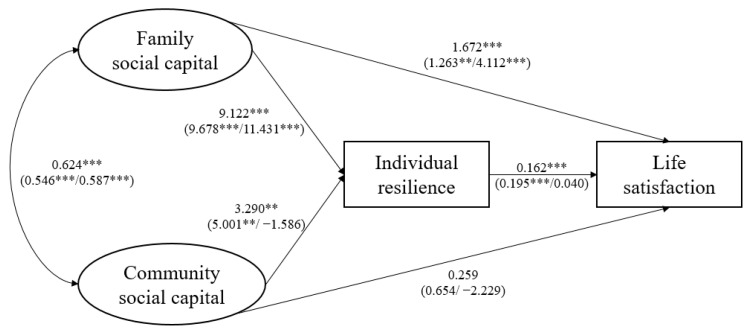
SEM results for life satisfaction. ** *p* < 0.05, *** *p* < 0.01.

Figure 2, Figure 3, Figure 4 and Figure 5 show the SEM results for four indicators of psychosocial adjustments of all cross-border students and all subgroups. Detailed results are listed in Appendix B. To enable interpretation, paths linking family and community social capital and outcome variables are also summarized in Table 2.

Supporting hypothesis 1a, family social capital had a significant, positive, and direct impact on cross-border students’ self-esteem, mental well-being, happiness, and life satisfaction. In addition, family social capital exerted significant indirect effects on cross-border students’ psychosocial adjustments through increasing individual resilience, consistent with hypothesis 2a. In total, one SD increase in family social capital led to increases in cross-border students’ self-esteem, mental well-being, happiness, and life satisfaction by 1.864, 5.613, 0.295, and 3.150 units, respectively.

As for the influence of community social capital, in contrast to hypothesis 1b, community social capital did not have significant direct effects on cross-border students’ self-esteem, mental well-being, happiness, or life satisfaction. Despite results partially supporting hypothesis 2b and indicating that community social capital could slightly increase cross-border students’ mental well-being and life satisfaction through individual resilience, community social capital did not have significant total effects on four indicators of cross-border students’ psychosocial adjustments.

Subgroup analysis produced several major results. First, family social capital had significant positive total effects on cross-border students’ self-esteem, mental well-being, happiness, and life satisfaction, both for single/no adversity and for multiple adversities. This finding provided evidence that family social capital acted as a significant promotive factor for cross-border students, no matter the levels of adversity they face. Second, cross-border students’ psychosocial adjustments indicators were more sensitive to changes in family social capital when faced with multiple adversities. This finding supported hypothesis 3a, and suggested that family social capital also served as a protective factor for cross-border students and played a special role when the level of adversity was high. Third, when there were multiple adversities (i.e., compared to single or no-adversity situations), family social capital exerted a more direct influence on cross-border students’ psychosocial adjustments. Fourth, contradictory to hypothesis 3b, community social capital had significant total effects on cross-border students’ mental well-being only when adversity level was low, and did not significantly influence their mental health and life satisfaction indicators when there were multiple adversities. Last, surprisingly, when there were multiple adversities, one SD increase in community social capital actually led to reductions in cross-border students’ self-esteem, mental well-being, happiness, and life satisfaction by 0.763, 1.592, 0.216, and 2.293 units, respectively, though these were not statistically significant. This finding suggested that community social capital might play completely opposite roles in shaping cross-border students’ psychosocial adjustments in the face of different levels of adversity.

Except for performing analysis on indicators of psychosocial adjustment separately, we also adopted an integrated model in which psychosocial adjustment was treated as a latent variable that was constructed by self-esteem, mental well-being, happiness, and life satisfaction. The results of integrated model (both measurement and structural) were listed in Appendice C and Appendice D. Paths linking family and community social capital and psychosocial adjustment are also summarized in Appendix E. The major findings of integrated model were consistent with Table 2. Family social capital mattered more than community social capital in promoting cross-border students’ psychosocial adjustments. Community social capital only enhanced cross-border students’ psychosocial adjustments through individual resilience in the case of no or single adversity. Moreover, when there were multiple adversities, community social capital was negatively associated with cross-border students’ psychosocial adjustments, though not significantly.

## 5. Discussion and Conclusions

Situated between internal and international migration, cross-border students are a population of mobility that is unique to the geopolitical context of Hong Kong. Existing migration studies have examined the drivers and consequences of internal and international migration, but little attention has been given to these cross-border students. Nearly 28,000 kindergarten, primary, and secondary school students reside in mainland China, and cross the border between mainland China and Hong Kong every day to attend school and receive education in Hong Kong. This study proposes a social capital and resilience framework to understand their daily border-crossing experience. This study also contributes to the literature by providing empirical evidence on the psychosocial adjustments of cross-border students. Drawing on a cross-sectional survey of 445 cross-border students, this study has three major findings.

First, family social capital has a significant direct influence on cross-border students’ self-esteem, mental well-being, happiness, and life satisfaction. Family social capital also indirectly promotes self-esteem, mental well-being, happiness, and life satisfaction of cross-border students through increasing their individual resilience. Consistent with existing studies that examine the mental health and well-being of children involved in internal and international migration (for example, [47,48]), our findings suggest that family social capital acts as a significant promotive factor for cross-border students’ positive psychosocial adjustments. 

Second, this study provides evidence that family social capital exerts stronger total influence on cross-border students when they suffer from multiple adversities. In other words, family social capital has greater influence on cross-border students when the levels of adversity are high. This finding suggests that social capital serves additionally as an important protective factor for cross-border students. Our findings further point out that, when the level of adversity is high, family social capital is more likely to work in a direct way to protect cross-border students’ positive psychosocial adjustments. One possible reason is that, when being exposed to higher risks or more adversity, parents may provide more emotional support to, pay more attention to, and spend more time with their children, coping with adversity by explaining why they have chosen cross-border schooling, helping children understand their sacrifices, and building closer bonds with children.

Third, different from family social capital, in this study, community social capital does not have significant total effects on cross-border students’ psychosocial adjustments. Community social capital, however, has variable effects on cross-border students’ psychosocial adjustments, depending on the level of adversity. When adversity levels are low, community social capital promotes cross-border students’ mental well-being, happiness, and life satisfaction through increasing their resilience. This finding is partially consistent with existing studies, which have documented the contributing roles played by community social capital in shaping migrants’ education and health outcomes [49,50]. With multiple adversities, community social capital is negatively associated, though not significantly, with cross-border students’ psychosocial adjustments. This may be caused by the context-specific nature of community social capital. As these cross-border students reside in mainland China, higher community social capital means closer bonding with mainland neighbors and a higher sense of belonging to mainland residential communities. In the presence of multiple adversities, the split between their caring mainland communities and their challenging cross-border schooling life may result in social withdrawal and identity confusion, leading to further deterioration in mental health and life satisfaction. One recent study suggests that building community social capital specific to the Hong Kong context, for example, participation in extra-curriculum activities sponsored by social service organizations and churches and friendships with local students in Hong Kong, enables “double-not” children’s positive psychosocial adjustments [41]. 

This study contributes to the literature in several ways. First, we propose a new conceptual framework synthesizing social capital theory and individual resilience. Even though the unit of analysis is individual, this framework highlights that individual resilience is subject to the influence of social relations and resources embedded in family and community, permitting cascades across the individual, the family, and the community and echoing a developmental systems approach to resilience [7,8]. The framework also enables us to explore how interrelated family and community social capital enhance individual resilience, which further increases cross-border students’ positive psychosocial adjustments. Second, this study provides updated empirical evidence for a more comprehensive understanding of cross-border students’ experiences, especially in terms of their mental health and psychological adjustments. Third, this study emphasizes the protective and promotive effects of family social capital, and reveals that community social capital may be context-specific and may work in a multidirectional way when the level of adversity varies.

This study has some limitations. First, given the nature of cross-sectional data used in this study, the mediation effects of individual resilience should be interpreted with caution. We cannot rule out the possibility that cross-border students with higher individual resilience may be more able to perceive parents’ sacrifices and struggle in supporting their education and, hence, may have closer relationships with their parents and higher reserves of family social capital. Next, despite our consideration of family and community social capital as two important factors for enhancing individual resilience and mental health and our measurement of them in different aspects with several items, social capital embedded in other intermediate spheres, such as classrooms and schools, was not addressed in this study. Support from peers and teachers may also help cross-border students overcome their difficulties and make positive psychosocial adjustments. Last, our survey was completed before the COVID-19 pandemic, and cross-border students may now be living in Hong Kong or may have encountered new difficulties during the time of pandemic. Further studies could follow a cohort of cross-border students, collect longitudinal data, and exploit causal analysis methods to establish more accurate and comprehensive knowledge of the development and transition of this special mobile demographic group.

Despite these limitations, the findings of this study carry important theoretical and practical implications. Theoretically, this study integrates social capital theory and resilience framework, and it provides evidence that levels of adversity matter for the building and functioning of individual resilience. Though this study has a focus on cross-border students’ mental health outcomes, the framework can be extended to other dimensions of their development, such as educational outcomes. This study also supports the idea that the role played by family and social capital varies depending on adversity level. Further studies should devote more effort to examine the threshold effects of adversity [42].

Practically, this study highlights the promotive and protective effects of family social capital, especially when adversity level is high. Parents of cross-border students are encouraged to provide more emotional support and build close relationships with their children to help them overcome challenges and make positive psychological adjustments. This study finds that the role played by community social capital is very context-specific. This finding also provides a clue for intervention by parents and social-service organizations. Both parents and cross-border students are encouraged to plan ahead and take the long view of cross-border students’ education and development. If cross-border students plan to settle down in Hong Kong after graduation, parents should help them build up community social capital that is specific to the Hong Kong context—for example, participating in extra-curricular activities in Hong Kong, visiting cultural museums to become familiar with Hong Kong, and forming interpersonal relationships in Hong Kong schools. Social-service organizations can also serve as bridges connecting the two different educational and political regimes by narrowing the information gap, providing more information on the Hong Kong educational system, and offering language tutoring programs tailored to the needs of cross-border students.

## Figures and Tables

**Table 1:** Descriptive statistics.

	Mean/Percentages	S.D.	Min	Max	N
**Psychosocial adjustments**					
Self-esteem	29.295	5.384	10	40	421
Mental well-being	51.440	13.550	14	70	414
Happiness	3.831	1.001	1	5	414
Life satisfaction	25.100	7.653	5	35	409
**Adversity**					
Total number of adversities experienced	1.975	2.996	0	14	400
**Resilience and social capital**					
*Individual resilience*					
Individual resilience score	75.233	21.508	15	105	400
*Family social capital*					
Parent–child interaction	4.661	2.294	0	8	440
Parental monitoring	20.190	4.927	8	32	426
Parents’ knowledge of children’s after-school whereabouts	15.027	4.616	4	20	438
Relationship with mother	43.315	7.851	17	60	406
Relationship with father	42.689	9.207	12	60	389
*Community social capital*					
Do neighbors care about you?	3.111	1.176	1	5	443
Social cohesion and trust among adults	15.868	3.803	5	25	431
Social cohesion and trust among children	17.026	4.033	5	25	428
Sense of belonging to the community	25.538	8.626	8	40	418
**Control variables**					
Male	52.871%		0	1	418
Age (years)	11.108	1.604	8	17	437
Father obtained associate degree or above	12.557%		0	1	438
Mother obtained associate degree or above	8.716%		0	1	436
Live with father	82.883%		0	1	444
Live with mother	95.045%		0	1	444
Duration of being cross-border student (years)	5.235	2.483	0	15.417	400

**Table 2:** Summary of paths linking family and community social capital and cross-border students’ psychosocial adjustments.

	Self-Esteem			Mental Well-Being			Happiness			Life Satisfaction		
	Total	SA	MA	Total	SA	MA	Total	SA	MA	Total	SA	MA
**Family social capital**												
*Direct effect*	1.209 ***	1.325 **	1.582 **	3.046 ***	2.410 **	7.298 ***	0.157 **	0.108 **	0.258 *	1.672 ***	1.263 **	4.112 ***
	(0.394)	(0.548)	(0.763)	(1.098)	(1.086)	(2.679)	(0.069)	(0.051)	(0.136)	(0.559)	(0.591)	(1.153)
*Indirect effect*												
→Through individual resilience	0.655 ***	0.591 **	0.576 **	2.567 ***	3.284 ***	1.087	0.138 ***	0.140 ***	0.154 ***	1.478 ***	1.886 ***	0.462
	(0.177)	(0.287)	(0.268)	(0.713)	(0.726)	(1.214)	(0.026)	(0.032)	(0.058)	(0.295)	(0.156)	(0.707)
**Total effect**	1.864 ***	1.916 ***	2.159 ***	5.613 ***	5.694 ***	8.386 ***	0.295 ***	0.248 ***	0.412 ***	3.150 ***	3.149 ***	4.574 ***
	(0.273)	(0.338)	(0.533)	(0.691)	(0.613)	(1.787)	(0.075)	(0.059)	(0.133)	(0.495)	(0.655)	(0.574)
**Community social capital**												
*Direct effect*	0.082	0.221	−0.685	0.699	0.317	−1.471	−0.008	0.103	−0.196	0.259	0.654	−2.229
	(0.237)	(0.571)	(0.955)	(1.043)	(1.036)	(2.19)	(0.105)	(0.112)	(0.220)	(0.541)	(0.708)	(1.376)
*Indirect effect*												
→Through individual resilience	0.216	0.283	−0.077	0.972 **	1.563 *	−0.121	0.048	0.074 *	−0.020	0.533 *	0.975 *	−0.064
	(0.145)	(0.235)	(0.137)	(0.427)	(0.856)	(0.258)	(0.03)	(0.040)	(0.038)	(0.274)	(0.515)	(0.128)
**Total effect**	0.298	0.503	−0.763	1.670	1.880 **	−1.592	0.040	0.177	−0.216	0.792	1.629	−2.293
	(0.328)	(0.493)	(0.956)	(1.236)	(0.938)	(2.225)	(0.127)	(0.138)	(0.228)	(0.712)	(1.059)	(1.426)
N	445	252	148	445	252	148	445	252	148	445	252	148

Notes: SA—single or no adversity; MA—multiple adversities. A total of 445 samples were included in the full sample analysis. Only 400 samples were included in the subgroup analysis due to missing values of adversity. Robust standard errors clustered at the school level are in parentheses. Indirect effects were tested using the bootstrapping method. * *p* < 0.1, ** *p* < 0.05, *** *p* < 0.01.

## Data Availability

The data presented in this study are available on reasonable request from the first author. The data are not publicly available due to ethical considerations.

## Appendix A

**Table A1:** Estimates of factor loadings from the measurement model.

	Unstandardized		Standardized
	(Standard Error)		
**Family social capital**			
Parent–child interaction	1.000 ^1^		0.740
Parental monitoring	1.729	(0.167)	0.596
Parents’ knowledge of children’s after-school whereabouts	1.383	(0.168)	0.509
Relationship with mother ^2^	2.831	(0.305)	0.604
Relationship with father ^2^	3.125	(0.363)	0.575
**Community social capital**			
Do neighbors care about you?	1.000 ^1^		0.634
Social cohesion and trust among adults	4.015	(0.310)	0.788
Social cohesion and trust among children	4.020	(0.329)	0.744
Sense of belonging to the community	9.627	(0.737)	0.835
**Overall goodness of fit** ^3^			
RMSEA	0.056		
CFI	0.972		
TLI	0.960		

Notes: ^1^ For each construct, factor loading of one indicator is conventionally fixed to unity for model identification. ^2^ Errors of this indicator were allowed to correlate according to modification indexes. ^3^ In STATA, SRMR is not reported because of missing values. All factor loadings are significant at *p* < 0.001.

## Appendix B

**Table A2:** Results of SEM of full and sub-group samples.

	Self-esteem			Mental Well-Being			Happiness			Life Satisfaction		
	Total	SA	MA	Total	SA	MA	Total	SA	MA	Total	SA	MA
**Psychosocial adjustments**												
Individual resilience	0.071 ***	0.061 *	0.051 **	0.285 ***	0.330 ***	0.098	0.015 ***	0.015 ***	0.014 ***	0.162 ***	0.195 ***	0.040
	(0.021)	(0.035)	(0.025)	(0.069)	(0.086)	(0.107)	(0.003)	(0.004)	(0.005)	(0.030)	(0.024)	(0.065)
Family social capital	1.209 ***	1.325 **	1.582 **	3.046 ***	2.410 **	7.298 ***	0.157 **	0.108 **	0.258 *	1.672 ***	1.263 **	4.112 ***
	(0.394)	(0.548)	(0.763)	(1.098)	(1.086)	(2.679)	(0.069)	(0.051)	(0.136)	(0.559)	(0.591)	(1.153)
Community social capital	0.082	0.221	−0.685	0.699	0.317	−1.471	−0.008	0.103	−0.196	0.259	0.654	−2.229
	(0.237)	(0.571)	(0.955)	(1.043)	(1.036)	(2.190)	(0.105)	(0.112)	(0.220)	(0.541)	(0.708)	(1.376)
Total adversity	−0.233 *	0.659	−0.166	0.101	−0.292	0.520	−0.072 ***	−0.073	−0.048 *	−0.015	0.826	0.085
	(0.123)	(0.675)	(0.168)	(0.379)	(0.914)	(0.351)	(0.022)	(0.080)	(0.027)	(0.127)	(0.829)	(0.198)
Male	1.076 ***	0.855	1.632 ***	1.201	1.322	−0.554	0.132	0.105	0.161	0.551	1.040	−1.115
	(0.319)	(0.574)	(0.385)	(1.164)	(1.298)	(2.018)	(0.113)	(0.113)	(0.230)	(0.739)	(0.763)	(1.427)
Age	0.144	0.483 **	−0.191	0.311	0.258	0.514	−0.048 *	−0.037	−0.025	−0.069	0.173	−0.466
	(0.095)	(0.188)	(0.225)	(0.249)	(0.323)	(0.554)	(0.026)	(0.025)	(0.053)	(0.127)	(0.124)	(0.355)
Father obtained associate	1.080 *	1.143	0.675	1.165	0.873	0.389	0.087	−0.162	0.405	0.003	0.252	−0.296
degree or above	(0.648)	(0.854)	(1.830)	(1.865)	(1.927)	(4.422)	(0.142)	(0.199)	(0.410)	(0.991)	(0.799)	(1.548)
Mother obtained associate degree	0.776	0.828	0.467	−0.805	−0.860	−2.034	0.197	0.219	0.193	0.850	−0.056	1.474
degree or above	(0.535)	(1.426)	(1.119)	(1.141)	(1.984)	(1.558)	(0.162)	(0.275)	(0.245)	(1.067)	(1.384)	(1.827)
Live with father	0.406	1.505	−1.345 *	−0.108	1.758	−6.042	−0.001	0.119	−0.268	0.208	−1.247	−0.792
	(0.594)	(1.004)	(0.786)	(1.767)	(2.641)	(4.091)	(0.187)	(0.181)	(0.222)	(0.818)	(1.389)	(1.254)
Live with mother	−1.835 **	−3.604 *	−1.206	−3.222	−5.498 **	−4.585	−0.266	−0.227	−0.601	−1.552	−1.304	−3.357
	(0.778)	(1.908)	(1.367)	(2.334)	(2.414)	(4.590)	(0.207)	(0.236)	(0.387)	(1.094)	(1.529)	(2.393)
Duration of being cross-border	0.171	0.145	0.256	0.277	0.100	0.894 *	0.008	0.024	−0.011	0.060	−0.183	0.856 ***
student	(0.143)	(0.173)	(0.167)	(0.182)	(0.178)	(0.540)	(0.015)	(0.020)	(0.021)	(0.116)	(0.137)	(0.260)
Constant	22.496 ***	20.433 ***	27.112 ***	27.194 ***	26.885 ***	39.965 ***	3.478 ***	3.256 ***	3.659 ***	14.342 ***	11.353 ***	25.700 ***
	(2.474)	(3.586)	(2.530)	(8.366)	(6.745)	(13.668)	(0.404)	(0.613)	(0.750)	(4.135)	(4.159)	(4.677)
**Individual Resilience**												
Family social capital	9.228 ***	9.736 ***	11.357 ***	9.018 ***	9.966 ***	11.074 ***	9.203 ***	9.628 ***	11.249 ***	9.122 ***	9.678 ***	11.431 ***
	(1.275)	(1.565)	(2.492)	(1.119)	(1.459)	(2.231)	(1.383)	(1.568)	(2.445)	(1.217)	(1.436)	(2.344)
Community social capital	3.039 **	4.657 *	−1.523	3.413 ***	4.745 *	−1.229	3.213 **	5.103 **	−1.446	3.290 **	5.001 **	−1.586
	(1.518)	(2.457)	(2.719)	(1.316)	(2.422)	(2.555)	(1.585)	(2.389)	(2.634)	(1.352)	(2.171)	(2.664)
Total adversity	−0.686 **	1.535	−0.150	−0.673 **	1.177	−0.162	−0.669 **	1.289	−0.172	−0.706 **	1.300	−0.208
	(0.293)	(2.845)	(0.496)	(0.314)	(2.814)	(0.507)	(0.310)	(2.794)	(0.491)	(0.293)	(2.756)	(0.486)
Male	−0.078	1.188	−0.820	0.341	1.970	−0.945	0.081	1.294	−0.871	0.454	1.493	−1.014
	(2.428)	(2.214)	(4.832)	(2.442)	(2.200)	(4.940)	(2.409)	(2.289)	(5.028)	(2.375)	(2.231)	(4.891)
Age	1.444 *	1.917	0.822	1.405 *	2.045 *	0.785	1.437 *	2.061 *	0.764	1.465 *	1.866 *	0.840
	(0.871)	(1.166)	(1.085)	(0.797)	(1.122)	(1.074)	(0.837)	(1.195)	(1.068)	(0.816)	(1.127)	(1.045)
Father obtained associate	−2.403	−5.129	−2.051	−2.890	−5.384	−2.085	−2.959	−5.454	−2.526	−2.661	−5.707	−2.300
degree or above	(2.245)	(3.529)	(4.815)	(1.843)	(3.411)	(4.541)	(2.065)	(3.504)	(4.633)	(2.068)	(3.875)	(4.676)
Mother obtained associate	1.209	2.170	−1.730	1.706	2.640	−0.843	1.777	2.262	−0.495	1.337	2.397	−0.966
degree or above	(2.896)	(4.387)	(5.544)	(3.108)	(4.664)	(5.396)	(3.108)	(4.458)	(5.570)	(3.008)	(4.371)	(5.231)
Live with father	−3.393	−2.723	−9.220 **	−3.702	−3.213	−9.177 **	−3.486	−2.202	−9.489 **	−3.331	−2.145	−9.391 **
	(2.916)	(3.010)	(4.550)	(2.937)	(2.994)	(4.483)	(2.811)	(3.266)	(4.376)	(2.695)	(2.880)	(4.598)
Live with mother	−18.594 ***	−22.718 **	−16.833 ***	−18.017 ***	−22.947 **	−16.421 ***	−17.941 ***	−22.948 **	−17.214 ***	−18.823 ***	−22.769 **	−17.097 ***
	(4.395)	(9.080)	(5.952)	(4.266)	(9.079)	(5.821)	(4.223)	(9.164)	(5.738)	(4.255)	(9.153)	(5.613)
Duration of being cross-border	0.793 *	0.628	1.384	0.698	0.556	1.263	0.763 *	0.622	1.373	0.750 *	0.587	1.363
student	(0.454)	(0.382)	(0.955)	(0.450)	(0.363)	(0.904)	(0.453)	(0.379)	(0.964)	(0.455)	(0.383)	(0.943)
Constant	77.297 ***	77.061 ***	80.982 ***	77.605 ***	76.206 ***	81.662 ***	76.796 ***	75.295 ***	82.286 ***	77.262 ***	77.480 ***	81.685 ***
	(13.315)	(17.951)	(9.523)	(12.859)	(17.982)	(10.000)	(13.488)	(18.396)	(10.094)	(12.655)	(17.674)	(9.716)
N	445	252	148	445	252	148	445	252	148	445	252	148

Robust standard errors clustered at the school level are in parentheses. * *p* < 0.1, ** *p* < 0.05, *** *p* < 0.01.

## Appendix C

**Table A3:** Estimates of factor loadings from the integrated measurement model.

	Unstandardized		Standardized
	(Standard Error)		
**Family social capital**			
Parent–child interaction	1.000 ^1^		0.667
Parental monitoring	1.841	(0.184)	0.571
Parents’ knowledge of children’s after-school whereabouts	1.659	(0.182)	0.549
Relationship with mother ^2^	3.441	(0.334)	0.663
Relationship with father ^2^	3.595	(0.382)	0.595
**Community social capital**			
Do neighbors care about you?	1.000 ^1^		0.635
Social cohesion and trust among adults	4.007	(0.308)	0.788
Social cohesion and trust among children	4.032	(0.328)	0.748
Sense of belonging to the community	9.556	(0.728)	0.831
**Psychosocial adjustments**			
Self-esteem	1.000 ^1^		0.734
Mental well-being	2.894	(0.181)	0.849
Happiness	0.173	(0.013)	0.683
Life satisfaction	1.437	(0.106)	0.742
**Overall goodness of fit** ^3^			
RMSEA	0.058		
CFI	0.957		
TLI	0.945		

Notes: ^1^ For each construct, factor loading of one indicator is conventionally fixed to unity for model identification. ^2^ Errors of this indicator were allowed to correlate according to modification indexes. ^3^ In STATA, SRMR is not reported because of missing values. All factor loadings are significant at *p* < 0.001.

## Appendix D

**Table A4:** Results of SEM in the integrated model.

	Psychosocial Adjustments			Individual Resilience		
	Total	SA	MA	Total	SA	MA
Individual resilience	0.092 ***	0.093 ***	0.056 ***	-	-	-
	(0.015)	(0.023)	(0.021)	-	-	-
Family social capital	1.113 ***	1.032 ***	1.957 ***	9.308 ***	10.532 ***	10.358 ***
	(0.308)	(0.253)	(0.558)	(1.128)	(1.527)	(2.240)
Community social capital	0.159	0.229	−0.720	3.166 **	4.175 *	−0.164
	(0.328)	(0.237)	(0.597)	(1.299)	(2.181)	(2.400)
Total adversity	−0.088	0.170	0.053	−0.692 **	1.010	−0.137
	(0.085)	(0.341)	(0.100)	(0.300)	(2.813)	(0.515)
Male	0.577	0.605	0.265	−0.076	1.592	−0.701
	(0.422)	(0.455)	(0.522)	(2.470)	(2.405)	(4.790)
Age	0.010	0.056	0.048	1.357	1.999 *	0.986
	(0.038)	(0.076)	(0.171)	(0.838)	(1.185)	(1.127)
Father obtained associate	0.456	0.102	0.482	−2.972	−5.697 *	−1.929
degree or above	(0.552)	(0.475)	(1.280)	(1.987)	(3.167)	(4.524)
Mother obtained associate degree	0.276	0.101	0.224	1.538	2.293	−0.987
degree or above	(0.340)	(0.708)	(0.542)	(2.985)	(4.473)	(5.101)
Live with father	0.029	0.162	−1.060	−3.789	−3.740	−7.953 **
	(0.532)	(0.772)	(0.865)	(3.144)	(3.311)	(4.034)
Live with mother	−1.238 **	−2.338 ***	−0.966	−17.397 ***	−24.001 ***	−14.637 ***
	(0.623)	(0.869)	(1.169)	(4.149)	(9.206)	(5.263)
Duration of being cross-border	0.085	0.023	0.278 ***	0.727	0.577	1.186
student	(0.054)	(0.048)	(0.078)	(0.449)	(0.378)	(0.888)
Constant	-	-	-	77.801 ***	78.452 ***	76.880 ***
	-	-	-	(13.553)	(18.998)	(10.451)
N	445	252	148	445	252	148

Robust standard errors clustered at the school level are in parentheses. * *p* < 0.1, ** *p* < 0.05, *** *p* < 0.01.

## Appendix E

**Table A5:** Summary of paths in the integrated model.

	Psychosocial Adjustments		
	Total	SA	MA
**Family social capital**			
*Direct effect*	1.113 ***	1.032 ***	1.957 ***
	(0.308)	(0.253)	(0.558)
*Indirect effect*			
→Through individual resilience	0.852 ***	0.983 ***	0.584 **
	(0.146)	(0.155)	(0.268)
**Total effect**	1.965 ***	2.016 ***	2.541 ***
	(0.241)	(0.183)	(0.392)
**Community social capital**			
*Direct effect*	0.159	0.229	−0.720
	(0.328)	(0.237)	(0.597)
*Indirect effect*			
→Through individual resilience	0.290	0.390 *	−0.009
	(0.138)	(0.230)	(0.135)
**Total effect**	0.449	0.619 *	−0.729
	(0.417)	(0.331)	(0.613)
N.	445	252	148

Notes: SA—single or no adversity; MA—multiple adversities. A total of 445 samples were included in the full sample analysis. Only 400 samples were included in the subgroup analysis due to missing values of adversity. Robust standard errors clustered at the school level are in parentheses. * *p* < 0.1, ** *p* < 0.05, *** *p* < 0.01.
